# An Account of a Fatal Case of Acute Negro Consumption, with the Appearances on Dissection

**Published:** 1829

**Authors:** John Bennett

**Affiliations:** Newport, Kentucky


ART. II.—*An account of a fatal case of Acute Negro Consumption, with the appearances on dissection.* By Dr. JOHN BENNETT, of Newport, Kentucky.

On the 26th of April last, Mr. H—— requested me to prescribe for his servant, a negro girl, aged 21 years, who had the usual symptoms of the vernal fever, peculiar to our section of the country. I prescribed a mercurial cathartic, with castor oil, and heard nothing more from the patient until the 2d of June, when, without visiting her, I directed them to repeat the cathartic. On the 4th, I was called to see her, and found her laboring under high fever, with pain in her head and back, tongue furred, and bowels constipated. I directed pills of calomel and scammony, to be given every two hours until they operated freely. They had the desired effect, and there was every indication of a speedy recovery. On the 26th, I was again called to see her, informed that her appetite had been good, and that she had indulged in eating every thing without restraint; that she had been permitted to visit the mineral well, distant one mile and half, and from thence she went to Newport, where she remained all night, and that on returning home, her disease was much aggravated. At this visit I found her somewhat emaciated, with a furred tongue, cough, expectoration of mucus, and soreness in the epigastric region. I directed a blister over the pit of the stomach, a restricted diet, and mercurial cathartics. I saw her again on the 30th, at which time her appetite was much impaired, she vomited frequently, and her cough had become troublesome; she also complained of slight pain in her left breast. From this date until her death, which occurred on the 14th of August, my attendance was pretty constant. During the time, Dr. Drake saw her once, and advised moderate bloodletting, the blue pill, and a dilute solution of tartarised antimony with tincture of digitalis. Throughout this stage of her disease, she complained of little or no pain, was generally cheerful, and on every visit, told me she was mending fast. Her tongue was clean, bowels regular, and her cough less troublesome. During the whole course of her illness, she expectorated but little, and that was a thin mucus. Her appetite continued tolerably good to the last, yet she was reduced to a mere skeleton. She often complained that worms were crawling in her throat and choaking her.

On the morning of the 15th of August, I was permitted to examine the body. On opening the cavity of the abdomen, I found a considerable quantity of fluid of a deep yellow colour, which I presume derived its hue from a mixture of bile; the liver was unusually large and of the same complexion; on passing the finger over its surface, it was found loaded with small tubercles; on cutting into its substance, no ulcerations were discovered. The gall bladder was considerably contracted, contained but a small quantity of bile, which was of a yellow colour. The spleen was much enlarged, its complexion a shade lighter than in the healthy state. On making a section of it, I found numerous small ulcerated spots, which contained a thin white matter. The thorax contained upwards of a pint of fluid, of the same consistence and colour with that of the abdomen. The whole substance of the lungs was tuberculated. The tubercles were small, hard and distinct, having in no place suppurated. The heart was small, soft, and flaccid. A circular hole was found in the pericardium, large enough to admit the point of the finger; this hole appears to have given a free circulation to the fluid from the cavity of the thorax to the heart, *et vice versa.*

Three tumors, containing matter not unlike new cheese, were found adhering to the larynx. They were of an oblong shape; the largest about two inches in length, and its diameter equal to that of the thumb of an adult. These tumors, I conjecture, caused the choaking sensation of which she complained, and which she attributed to worms. The cellular substance of the abdomen and chest was tinged yellow; yet her eyes exhibited nothing of a jaundiced appearance; indeed, up to the period of her death, they bore that peculiar pearly whiteness, so uniformly observed in pulmonary consumption.

Among the morbid affections under which this patient labored, it is evident that jaundice must have constituted one; yet, why the eyes exhibited no tinge, I am unable to say. Is this exemption peculiar to the negro? I am inclined to think it is. I have no recollection of ever having met with a case of jaundiced eyes in a negro, though I have often met with well marked cases of hepatic derangement.

I presume that negro consumption is scrofulous tuberculation of the liver and lungs. A diseased action of the serous membranes simultaneously take place, and a consequent effusion of water into the thoracic and abdominal cavities. This effusion may destroy the patient before the tubercles have time to coalesce and suppurate, hence we may account for the mere mucous expectoration which we so constantly meet with in negro consumption.

So far as my observation extends this disease is generally called into activity, by catarrh, bilious fever, measles, or some other acute disease. Thus in the case just reported, the patient was attacked in April with remittent fever, which, with subsequent exposure and a full diet, no doubt, brought into action her tuberculous predisposition.

I will only add, that if I am not mistaken, I have seen the appearance of scrophulous tumours on the neck and other external parts of the body, followed by a great mitigation of the symptoms of internal tuberculation, and consequently the life of the patient prolonged.

*August* 16, 1829.

